# Systematic identification of non-coding RNA 2,2,7-trimethylguanosine cap structures in *Caenorhabditis elegans*

**DOI:** 10.1186/1471-2199-8-86

**Published:** 2007-09-29

**Authors:** Dong Jia, Lun Cai, Housheng He, Geir Skogerbø, Tiantian Li, Muhammad Nauman Aftab, Runsheng Chen

**Affiliations:** 1 Bioinformatics Laboratory, Institute of Biophysics, Chinese Academy of Sciences, Beijing, 100101, China; 2 Shanxi Agricultural University, Taigu, Shanxi, 030801, China; 3 Bioinformatics Research Group, Key Laboratory of Intelligent Information Processing, Institute of Computing Technology, Chinese Academy of Sciences, Beijing, 100080, China; 4 Graduate School of the Chinese Academy of Sciences, Beijing 100039, China

## Abstract

**Background:**

The 2,2,7-trimethylguanosine (TMG) cap structure is an important functional characteristic of ncRNAs with critical cellular roles, such as some snRNAs. Here we used immunoprecipitation with both K121 and R1131 anti-TMG antibodies to systematically identify the TMG cap structures for all presently characterized ncRNAs in *C. elegans*.

**Results:**

The two anti-TMG antibodies precipitated a similar group of the *C. elegans *ncRNAs. All snRNAs known to have a TMG cap structure were found in the precipitate, indicating that our identification system was efficient. Other ncRNA families related to splicing, such as SL RNAs and Sm Y RNAs, were also found in the precipitate, as were 7 C/D box snoRNAs. Further analysis showed that the SL RNAs and the Sm Y RNAs shared a very similar Sm binding site element (AAU_4–5_GGA), which sequence composition differed somewhat from those of other U snRNAs. There were also 16 ncRNAs without an Sm binding site element in the precipitate, suggesting that for these ncRNAs, TMG formation may occur independently of Sm proteins.

**Conclusion:**

Our results showed that most ncRNAs predicted to be transcribed by RNA polymerase II had a TMG cap, while those predicted to be transcribed by RNA plymerase III or located in introns did not have a TMG cap structure. Compared to ncRNAs without a TMG cap, TMG-capped ncRNAs tended to have higher expression levels. Five functionally non-annotated ncRNAs also have a TMG cap structure, which might be helpful for identifying the cellular roles of these ncRNAs.

## Background

The 2,2,7-trimethylguanosine (TMG) cap structure was first found at the 5'-end of low-molecular-weight nuclear RNAs, such as U1 small nuclear RNAs (snRNA), U2 snRNA, U5 snRNA and U3 small nucleolar RNAs (snoRNAs) from *Novikoff *hepatoma cells (reviewed in [1]). In nematodes and some other metazoans, 5'-end TMG caps have been extensively characterized for *trans*-spliced mRNAs and functional non-coding RNAs (ncRNAs), such as U1, U2, U4 and U5 snRNAs, and for some of the snoRNAs and spliced leader (SL) RNAs [2]. All TMG-capped snRNAs are transcribed by RNA polymerase II.

In mammals, hypermethylation of the m^7^G cap of the U1, U2, U4, and U5 snRNAs to a TMG cap occurs in the cytoplasm, and depends on some of the Sm core proteins [3,4]. Unlike the assembly of the spliceosomal snRNPs, which take place in the cytoplasm, biogenesis of the vertebrate snoRNPs takes place in the nucleolar compartment, and hypermethylation of the TMG-capped snoRNAs is not associated with Sm proteins [5-7]. In yeast, U1, U2, U4, and U5 snRNAs also have a TMG cap structure [8] and hypermethylation of the cap structure is dependent on the Sm proteins [9,10], and a conserved methyltransferase, which is essential for hypermethylation of the m^7^G caps of snRNAs and snoRNAs[11]. Although the cellular function of the TMG cap is not clear, it is believed that the trimethylguanosine caps are necessary for the snRNAs to fulfill their cellular functions [12]. The TMG cap is an important component of the nuclear localization signal of U snRNPs, and there is evidence that core U-snRNPs without a TMG structure cannot be imported into the nucleus [13,14].

Biochemical determination of a TMG cap structure has traditionally required several experimental steps [15], and antibodies that recognize specific structures has therefore greatly facilitated screening for ncRNAs with TMG caps. Capped ncRNAs have been purified from total cellular RNA of vegetative microplasmodia by preparative immunoprecipitation with anti-TMG antibodies [16,17]. These antibodies specifically react with TMG-capped RNAs such as U1, U2 and U4 snRNAs. This can also be used to isolate snRNPs in one step from nuclear extracts of eukaryotic cells by affinity chromatography on a preparative scale [18].

Several strategies have been endeavored to detect or discover novel ncRNAs, including both experimental and computational screening [19-22]. Results from the recent tiling microarray studies also indicate that far larger portions of the eukaryote transcriptome than formerly believed are actually transcribed in the form of non-coding transcripts [23]. Systemic examination of the 5'-end TMG cap structures at the novel ncRNAs would be useful contribution towards decoding the cellular functions of these transcripts. Here we combined immunoprecipitation and an ncRNA microarray [24] to identify 5'-end TMG cap structures in most of the presently characterized ncRNAs in *C. elegans *[19], and analyzed important characters associated TMG-capped ncRNAs, such as function, biogenesis and expression.

## Results

### ncRNAs precipitated by the anti-TMG antibodies

**Figure 1:**
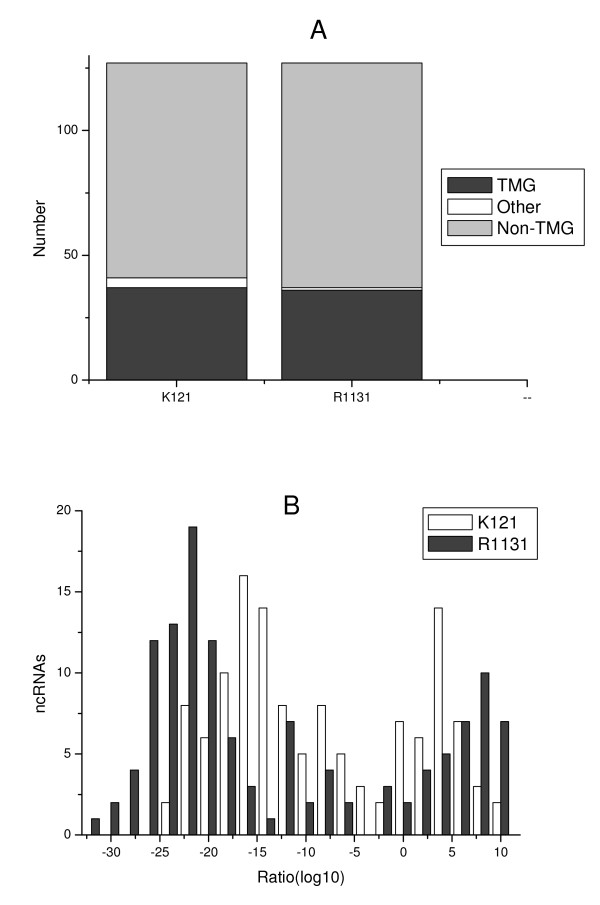
**Precipitation efficiency of K121 and R1131 anti-TMG antibodies **. (A) The number of ncRNAs that were assigned into TMG-capped group (TMG) or non-TMG-capped group (Non-TMG), or not assigned to either group (Other) by the K121 and R1131 anti-TMG antibodies. (B) Fluorescence intensity ratio distribution for the R1131 or K121 antibodies.

We first filtered and immunoprecipitated worm total RNA with the K121 anti-TMG antibody. However, after having been made aware that this antibody may have a reduced specificity for TMG caps and has shown some cross-reactivity with mono-methylated cap structures [25,26], we repeated all subsequent experiments with the R1131 antibody [17]. RNAs in both precipitate and supernatant obtained with both antibodies were extracted and reversely transcribed. The cDNA from precipitate and supernatant was labeled with Cy3 or Cy5, respectively, and hybridized to a microarray with probes for 127 ncRNAs [24]. For each of the 127 ncRNAs, the intensities of precipitation sample and supernatant sample were examined. Most of the ncRNAs had a clear tendency to occur either in the precipitation or in the supernatant sample. With a few exceptions, the two TMG antibodies precipitated the same ncRNAs, suggesting that both are able to effectively distinguish TMG capped ncRNAs from ncRNAs with other 5'-end structures (see below and Figure 1A). However, the data also indicated somewhat different specificities. For the snRNA U6, which have a simple γ-methylguanosine cap, a larger partition of this RNA was precipitated by K121 antibody (intensity ratio 0.55) than by R1131 antibody (intensity ratio 0.022; See Additional file 1). There was also a tendency towards precipitate and supernatant obtained with R1131 showing higher and lower intensity ratios, respectively, compared to those obtained with K121 (Figure 1B).

To get an estimate of the baseline expression, the Cy3 (or Cy5) intensity data from 8 repeats of mixed-stage worms were examined. Overall, the probability of the intensity of an ncRNA in a single repeat deviating 50% from the average was estimated to be 10%. We therefore used an intensity ratio (precipitation/supernatant) of 1.5 as a threshold for identifying TMG-capped RNAs, as at this threshold the false positive rate would be only 10%, and no more than two of the 127 ncRNAs studied would be falsely assigned TMG capped (or not). At this threshold, the K121 antibody precipitated 37 ncRNAs whereas R1131 precipitated 36 of these (Figure 1A and Additional file 1). As the R1131 antibody is known to have higher specificity for the TMG cap structure, the 36 ncRNAs that were precipitated by this antibody were assigned as TMG-capped for further analysis (Table 1).

**Table 1:** Intensity ratios (precipitation/supernatant) of 36 ncRNAs assigned to TMG-capped group by R1131

**Function**	**Name**	**R1131**	**K121**	**Function**	**Name**	**R1131**	**K121**
snRNA U1	CeN1-4	36.547	3.528	C/D snoRNA	CeN120	5.710	1.920
	CeN1-5	32.501	4.514		CeN30	4.435	3.609
	CeN1-1	21.351	4.226		CeN5	4.161	10.919
snRNA U2	CeN18	19.514	4.931		CeN22	3.788	4.177
snRNA U4	CeN2-2	6.481	5.236		CeN27	3.540	3.192
snRNA U5	CeN3-2	24.326	2.749		CeN122	1.730	1.056
	CeN3-5	19.875	2.757	U3 snoRNA	CeN26-1	3.222	2.323
	CeN3-6	15.115	1.503	Sm Y RNA	CeN31	34.423	6.568
snRNA SL2	CeN7	17.540	2.887		CeN112	29.063	2.726
	CeN12	14.904	4.494		CeN25-4	18.068	2.340
	CeN16-1	10.094	2.043		CeN25-1	15.759	2.693
	CeN16-3	9.829	2.525		CeN32	13.197	1.267
	CeN20	9.524	1.199		CeN115	6.440	3.758
	CeN19	7.870	2.492	Other	CeN37	28.436	7.172
	CeN16-4	7.762	3.218		CeN21-1	20.062	12.289
	CeN6	6.915	3.316		CeN35	14.605	9.307
	CeN8-1	5.237	1.574		CeN29	13.645	4.423
SnRNA SL1	CeN116	1.963	1.759		CeN23-1	9.368	3.196

Detailed studies the 5'-end structures of *C. elegans *ncRNA have apparently not been carried out, but all snRNAs known to be TMG-capped in other organisms [1] were assigned as TMG-capped in our analysis. Similarly, U6 snRNA, known to have a γ-methylated cap structure [1] was assigned to the non-TMG-capped group. Transcripts in ncRNA families known or assumed to be related to splicing, such as the SL RNAs and the recent identified Sm Y RNAs (snlRNAs in [19]) in *C. elegans *[24,27,28], were assigned to the TMG-capped group. There were also 7 C/D box snoRNAs in TMG-capped group, including U3 snoRNA, which has been shown to have a TMG cap in other organisms [7]. There were also 5 functionally non-annotated ncRNAs in the TMG-capped group. None of H/ACA box snoRNAs were detected in TMG-capped group.

### Most ncRNAs with an Sm binding site element are TMG-capped

**Figure 2:**
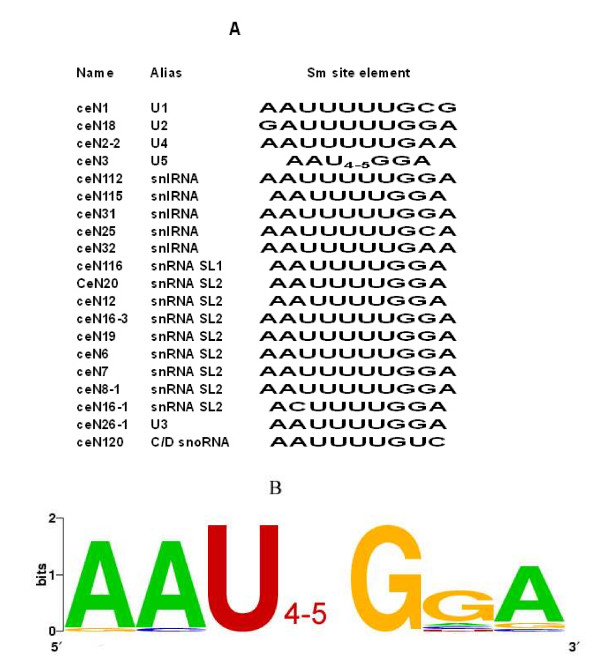
**Potential Sm binding site elements of TMG-capped ncRNAs**. (A) The potential Sm binding site elements of *C. elegans *TMG-capped ncRNAs, and (B) their consensus sequence.

The snRNAs are critical components of the splicesome, whose role is to remove introns from exons of immature mRNAs. In eukaryotes, Sm binding site elements (RAU_4–6_G) have been found in most snRNAs, such as U1, U2, U4 and U5 [29,30] and in yeast, maturation of the U3 snoRNA is dependent on some of the Lsm-proteins [31]. In nematodes, spliced leader (SL) RNAs, which participate in the processing of operonic genes, also have Sm binding site elements [32]. Binding of Sm proteins is thought to be a prerequisite for hypermethylation of the m^7^G cap to 2,2,7-trimethylguanosine by the Tgs1 methyltransferase [33]. The TMG cap and the associated Sm proteins provide the nuclear localization signal for the cytoplasmic core snRNP [33], suggesting that a TMG cap structure is always linked to the presence of an Sm binding site element. In our results, there were 22 ncRNAs that had a potential Sm binding site element, 19 of which were in the TMG-capped group. Other ncRNA families possibly related to splicing, such as the Sm Y RNAs, were also found in TMG-capped group. The potential Sm binding site elements found in these worm ncRNAs (Figure 2A) were slightly different from the consensus sequence established for other organisms (RAU_4–6_G) [29,30]. The Sm binding site sequence differed somewhat among the U1, U2 and U4 snRNAs, whereas U5 snRNA, U3 snoRNA and the SL RNAs and the Sm Y RNAs had very similar potential Sm binding site elements (consensus sequence AAU_4–5_GGA; Figure 2B).

There were also two TMG-capped C/D box snoRNA (U3 and CeN120) that contained potential Sm binding site elements. Of the remaining 91 ncRNAs, only two (CeN117 and CeN80) contain a similar pattern, and CeN117 was precipitated by K121 anti-TMG antibody with intensity ratios (precipitation/supernatant) above the threshold. There were also 16 ncRNAs without Sm binding site element that were enriched in the TMG-antibody precipitated fraction, indicating that hypermethylation of the m^7^G cap to 2,2,7-trimethylguanosine may occur independently of the Sm proteins.

### TMG cap status and transcription

**Figure 3:**
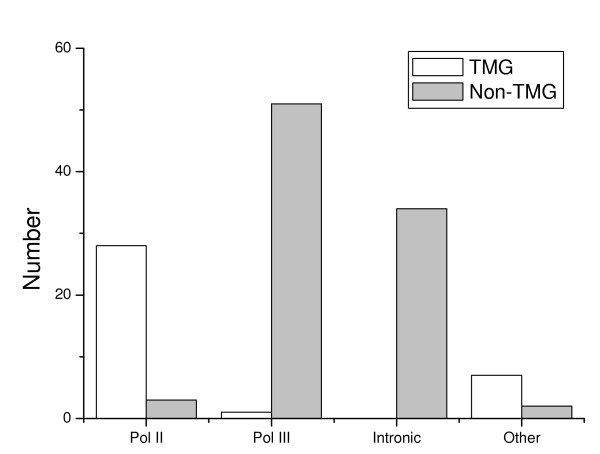
**Cap structure and predicted mode of biogenesis**. The ncRNAs were divided into four groups: those predicted to be transcribed by RNA polymerase II (Pol II) or III (Pol III), those located in introns with no evident upstream motifs (Intronic), and those with intergenic loci with no distinct upstream motifs (Other). The numbers of TMG-capped and non-TMG-capped ncRNAs were shown for each group.

Primary transcripts synthesized by RNA polymerase II are modified by addition of a 7-methylguanosine residue to their triphosphate 5'-end shortly after emergence from the polymerase. The 7-methylguanosine "caps" of small nuclear and small nucleolar RNAs are subsequently hypermethylated to a 2,2,7-trimethylguanosine cap. We therefore analyzed the relationship between TMG cap structure and the predicted mode of biogenesis for ncRNAs in *C. elegans*. Based on upstream motifs and other characteristics, ncRNAs in *C. elegans *have been divided into four groups [19], of which the first two were assumed to be transcribed by RNA polymerase II or III, respectively. The third group comprised ncRNAs processed from spliced introns with no evident upstream motif, and the fourth group was composed of other ncRNAs for which no predictions could be made. Twenty-eight of the 32 ncRNAs predicted to be transcribed by polymerase II [19], have a TMG-cap structure, the exceptions being four C/D box snoRNAs (CeN117, CeN121, CeN28 and CeN33), two of which were precipitated by K121 antibody. On the other hand, only one of 52 ncRNAs predicted to be transcribed by RNA polymerase III were assigned to the TMG-capped group. All the 34 intronic snoRNA without upstream motifs were also in the non-TMG-capped group. Eight of 9 intergenic ncRNA loci with no distinct upstream motif were in TMG-capped group, whereas one (CeN113) had intensity ratios (precipitation/supernatant) around 1.0 and the last one was assigned to the non-TMG group (Figure 3).

### Expression patterns of ncRNAs in TMG-capped group

**Figure 4:**
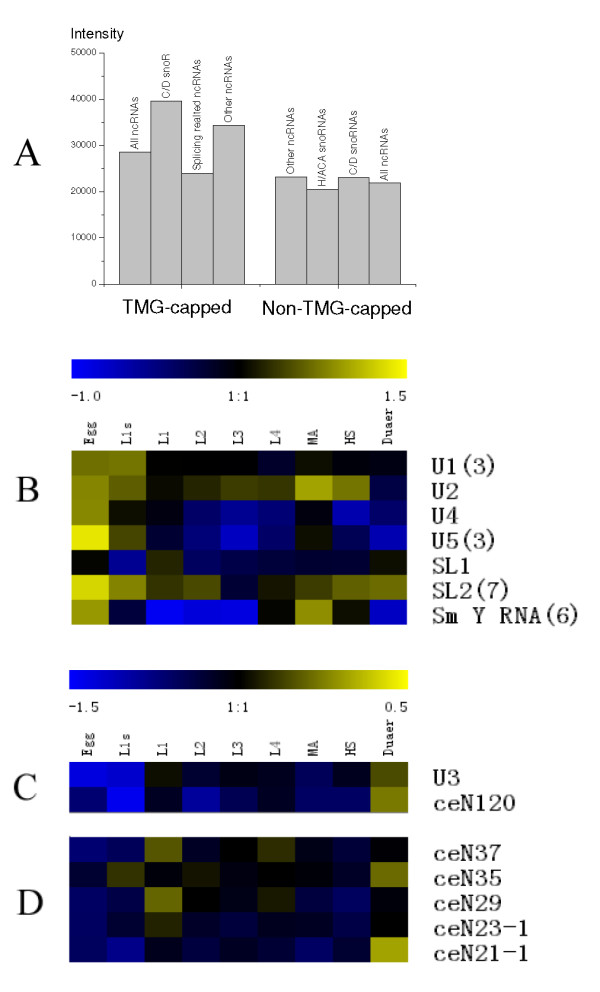
**Expression profiles of capped and non-capped of ncRNAs**. Expression levels for ncRNAs assigned to the TMG-capped and non-TMG-capped groups. The expression levels are given as the mean Cy3 or Cy5 fluorescence intensity for eight replicates. (B-D) Expression profiles of (B) the splicing related ncRNAs, (C), two C/D snoRNAs with Sm binding sites in the TMG-capped group, and (D) five uncharacterized C/D snoRNAs in the TMG-capped group during 7 development stages (Egg, L1 start (L1s), L1, L2, L3, L4 and MA), heatshock (HS) and dauer (Duaer). (The numbers in parenthesis in (B) indicate numbers of corresponding ncRNAs.)

We next reanalyzed previous microarray expression data for the *C. elegans *ncRNAs [24] with respect to cap structure. The TMG-capped ncRNAs were on average more highly expressed than non-TMG-capped ones. There were 7 C/D box snoRNAs with a TMG-cap structure, including snoRNA U3, and these TMG-capped C/D box snoRNAs had much higher expression levels than other snoRNAs (Figure 4A).

The expression patterns of the TMG-capped ncRNAs mainly fall into two groups, one containing splicing related ncRNAs (Figure 4B) and the other containing box C/D snoRNAs and functionally unannotated ncRNAs (Figures 4C and 4D). Splicing related ncRNAs had relatively lower expression levels than those in the other groups, but displayed greater fluctuations in expression levels over the developmental stages and stimulated conditions. The two C/D snoRNAs containing an Sm site element had very similar expression patterns, and showed significantly low expression at the earlier development stages and significantly high expression in dauer (Figure 4C). The other 4 C/D snoRNAs in TMG-capped group had relative low expression levels, and most displayed little variation in expression during development (the exception being CeN5 which was highly expressed in L1 start) [24].

There were also 5 non-annotated ncRNAs in the TMG-capped group. None of these ncRNAs have an Sm site element, however, their expression patterns are very similar to those of the two C/D snoRNAs in the TMG-capped group containing Sm binding site elements (Figures 4D).

## Discussion

The 2,2,7-trimethylguanosine cap structure is conserved in eukaryotes, and was first found at 5'-end of some snRNAs [15]. Antibodies against 2,2,7-trimethylguanosine can react with intact TMG-capped snRNPs in various organisms [18]. Here, our results showed that antibodies against 2,2,7-trimethylguanosine works well in *C. elegans*, and can be used to screen for TMG-capped RNAs.

Previous studies have shown that the R1131 antibody has a higher specificity for the TMG cap structure than the K121 antibody, and that the latter has shown some cross-reactivity with mono-methylated cap structures [25,26]. In our results, the R1131 antibody showed high affinity than the K121 antibody. There were more ncRNAs with intensity ratios (precipitation/supernatant) around 1.0 for the K121 antibody than those for R1131, indicating a lower efficiency for K121 in precipitation of TMG-capped ncRNAs than R1131 antibody. In general, precipitate and supernatant obtained with R1131 showed higher and lower intensity ratios, respectively, compared to those obtained with K121. This also possibly indicated that the R1131 has a higher affinity for TMG cap structures than the K121 at equal antibody concentrations.

In our previous work, the library construction procedure included a step to distinguish between 5'-end capped and non-capped RNAs. The probability of an ncRNA being 5'-end capped or not was determined [19]. The statistical model only partially detected the 5'-end capped ncRNAs, and could not distinguish between different kinds of 5'-end caps. Immunoprecipitation used here identified all ncRNAs previously found to be TMG-capped in other eukaryotes. Using immunoprecipitation and ncRNA chip strategies, of total 127 examined ncRNAs, we reassigned or refined the cap structure status previously assigned [19] for 11 ncRNAs.

With the exception of snRNA U6, which have a simple γ-methylguanosine cap in other studied organisms, all the spliceosomal snRNAs have a TMG cap structure [1,30]. In organisms where snRNA transcription has been investigated, U6 snRNA is always transcribed by RNA polymerase III [34,35], while the other snRNAs are transcribed by RNA polymerase II [36]. It is believed that the TMG cap hypermethylation of snRNAs is essential for the mature snRNAs return to nucleus [12]. Our results were consistent with the hypothesis that TMG capped ncRNAs are transcribed by RNA polymerase II in the sense that almost all ncRNAs detected to have a TMG cap structure had previously been predicted to be transcribed by RNA polymerase II [37].

All previously characterized TMG capped snRNAs, SL RNAs or snoRNAs have a potential Sm binding site elements suggesting that binding to Sm proteins may be a prerequisite for hypermethylation of the m^7^G cap to 2,2,7-trimethylguanosine by the Tgs1 methyltransferase [33]. Another conserved sequence element, the box C/D motif, has also been shown to direct snoRNA 5'-end m^7^G hypermethylation [38]. Our results further showed that also ncRNAs lacking both distinct Sm binding sites and box C/D elements had an TMG-cap structure suggesting that there may exist other pathways for hypermethylation of the m^7^G cap to 2,2,7-trimethylguanosine.

## Conclusion

Combining immunoprecipitation and ncRNA microarray strategies, we found that 36 ncRNAs in *C. elegans *were specifically precipitated by anti-2,2,7-trimethylguanosine antibodies. As anticipated, ncRNAs enriched by anti-TMG antibody mainly fall into certain functional categories and certain biogenesis group. Almost all ncRNAs containing an Sm binding site also had a TMG cap. Our results also showed that 16 ncRNAs in TMG-capped group did not have an Sm binding site element. Almost all ncRNAs in the TMG-capped group had been predicted to be transcribed by RNA polymerase II, whereas most ncRNAs in the non-TMG-capped group were assumed to be transcribed by RNA polymerase III or spliced from introns. Six functionally non-annotated ncRNAs were also found in the TMG-cap group, which suggests that these ncRNAs might also be transcribed by RNA polymerase II.

## Methods

### Immunoprecipitation of TMG-capped RNAs

Immunoprecipitations were done as described by Terns *et al*. [39] with some modifications. Briefly, total RNAs were extracted from mixed stage N2 worms, followed by several steps to remove mRNA, rRNA, and tRNA [19]. The remaining RNAs and K121 monoclonal anti-TMG antibody (Santa Cruz) or R1131 polyclonal anti-TMG antibody [17] (Synaptic System) were incubated in Binding Buffer (1% TritonX-100, 150 mM NaCl, 2 mM EDTA pH8.0, 20 mM Tris-HCl pH8.0) overnight at 4°C with continuous shaking. The RNA-antibody complexes were coupled to Protein G PLUS-Agarose Beads (Santa Cruz) for 2 hours at 4°C gentle rotation, and the supernatant and precipitate (beads) were separated by a gentle centrifugation. RNAs in the supernatant were extracted using Trizol (Invitrogen). The precipitated pellets were washed four times with cold Low Salt Wash Buffer (1% SDS, 1% TritonX-100, 2 mM EDTA, 150 mM NaCl, 20 mM Tris-HCl pH8.0), once with High Salt Wash Buffer (1% SDS, 1% TritonX-100, 2 mM EDTA, 500 mM NaCl, 20 mM Tris-HCl pH8.0) and twice with TE Buffer (pH 7.8). The final pellets were re-suspended in the Elution Buffer (1% SDS, 0.1 M NaHCO_3_), incubated for 5 min at 4°C with continuous inversion. After a gentle centrifugation, the RNAs in the supernatant were extracted from the Elution Buffer by using Trizol. The RNAs from both samples were used for the microarray strategies.

### ncRNA microarray procedures

The ncRNA microarray design has been described previously [24]. After immunoprecipitation, RNA samples were dephosphorylated with calf intestine alkaline phophatase (Fermentas), and ligated to the 3' adaptor oligonucleotide by T4 RNA ligase (Fermentas). The ligated ncRNAs were reverse transcribed using an oligonucleotide complementary to the 3' adaptor. The cDNAs from the supernatant and precipitation fractions were labeled with Cy5 and Cy3 respectively, using the Fermentas Amino Allyl cDNA labeling kit. The labeled cDNAs were combined and hybridized to the microarrays. The raw data were normalized and processed using the MIDAS (TIGR TM4) software as previously described [24].

## Authors' contributions

DJ and HH performed the experiments of immunoprecipitations and ncRNA microarray, interpreted the results. LC and GS analyzed the results and drafted the manuscript. MNA and TL participated in the design of the study and assisted in editing the manuscript. RC directed the design of the study and assisted in editing the manuscript. All the authors read and approved the final manuscript.

## Supplementary Material

Additional file 1 **Fluorescence intensity ratios (precipitation/supernatant) of 127 ncRNAs**. For each of 127 ncRNAs, fluorescence intensity ratios (precipitation/supernatant) were examined for both of K121 and R1131 anti-TMG antibodies. Click here for file
