# Supplementary material for: Systematic identification of non-coding RNA 2,2,7-trimethylguanosine cap structures in Caenorhabditis elegans
Source: BMC Mol Biol. 2007 Sep 29;8:86. doi: 10.1186/1471-2199-8-86 (PMC2200864; doi:10.1186/1471-2199-8-86)
Supplement: Additional file 1 — Fluorescence intensity ratios (precipitation/supernatant) of 127 ncRNAs. For each of 127 ncRNAs, fluorescence intensity ratios (precipitation/supernatant) were examined for both of K121 and R1131 anti-TMG antibodies. [file 1471-2199-8-86-S1.doc]

**Fluorescence intensity ratios (precipitation/supernatant) of 127 ncRNAs**

The fluorescence intensity ratios (precipitation/supernatant) were listed for both of K121 and R1131 anti-TMG antibodies. Each ncRNA was assigned into TMG-capped group (TMG:Y) or non-TMG-capped group (TMG:N), or not assigned to either group ((TMG:U).

| Name | R1131 | TMG | K121 | TMG | Function |
| --- | --- | --- | --- | --- | --- |
| ceN1-4 | 36.54651 | Y | 3.527825 | Y | snRNA U1 |
| ceN31 | 34.42291 | Y | 6.568258 | Y |  |
| ceN1-5 | 32.50062 | Y | 4.514435 | Y | snRNA U1 |
| ceN112 | 29.06306 | Y | 2.726469 | Y |  |
| ceN37 | 28.43624 | Y | 7.172005 | Y |  |
| ceN3-2 | 24.32563 | Y | 2.749257 | Y | snRNA U5 |
| ceN1-1 | 21.35142 | Y | 4.225764 | Y | snRNA U1 |
| ceN21-1 | 20.06238 | Y | 12.28914 | Y |  |
| ceN3-5 | 19.87498 | Y | 2.756849 | Y | snRNA U5 |
| ceN18 | 19.51424 | Y | 4.930648 | Y | snRNA U2 |
| ceN25-4 | 18.06798 | Y | 2.340118 | Y |  |
| ceN7 | 17.53976 | Y | 2.886539 | Y | snRNA sls-2 |
| ceN25-1 | 15.75861 | Y | 2.69324 | Y |  |
| ceN3-6 | 15.11486 | Y | 1.503486 | Y | snRNA U5 |
| ceN12 | 14.904 | Y | 4.4944 | Y | snRNA sls-2 |
| ceN35 | 14.60473 | Y | 9.306566 | Y |  |
| ceN29 | 13.64526 | Y | 4.423215 | Y |  |
| ceN32 | 13.19664 | Y | 1.267167 | Y |  |
| ceN16-1 | 10.09372 | Y | 2.042581 | Y | snRNA sls-2 |
| ceN16-3 | 9.829495 | Y | 2.524915 | Y | snRNA sls-2 |
| ceN20 | 9.523657 | Y | 1.198957 | U | snRNA sls-2 |
| ceN23-1 | 9.368329 | Y | 3.196388 | Y |  |
| ceN19 | 7.870134 | Y | 2.492277 | Y | snRNA sls-2 |
| ceN16-4 | 7.762227 | Y | 3.218249 | Y | snRNA sls-2 |
| ceN6 | 6.914872 | Y | 3.315851 | Y | snRNA sls-2 |
| ceN2-2 | 6.481433 | Y | 5.23626 | Y | snRNA U4 |
| ceN115 | 6.43982 | Y | 3.758284 | Y |  |
| ceN120 | 5.709862 | Y | 1.919818 | Y | snoRNA C/D |
| ceN8-1 | 5.237094 | Y | 1.574157 | Y | snRNA sls-2 |
| ceN30 | 4.435326 | Y | 3.608541 | Y | snoRNA C/D |
| ceN5 | 4.161163 | Y | 10.91934 | Y | snoRNA C/D |
| ceN22 | 3.788193 | Y | 4.177282 | Y | snoRNA C/D |
| ceN27 | 3.540102 | Y | 3.19213 | Y | snoRNA C/D |
| ceN26-1 | 3.221503 | Y | 2.323333 | Y |  |
| ceN116 | 1.963169 | Y | 1.758818 | Y | snRNA SL1 |
| ceN122 | 1.729529 | Y | 1.056392 | U | snoRNA C/D |
| ceN117 | 0.837604 | N | 1.558734 | Y | snoRNA C/D |
| ceN113 | 0.508537 | N | 0.772362 | U | snoRNA C/D |
| ceN33 | 0.487604 | N | 0.851687 | U | snoRNA C/D |
| ceN14 | 0.483918 | N | 0.272892 | N | snoRNA C/D |
| ceN74-2 | 0.41557 | N | 1.372773 | Y |  |
| ceN121 | 0.355857 | N | 2.952391 | Y | snoRNA C/D |
| ceN67 | 0.248338 | N | 0.370588 | N | snoRNA H/ACA |
| ceN85 | 0.167638 | N | 0.302899 | N | snoRNA H/ACA |
| ceN105 | 0.164557 | N | 0.054166 | N | snoRNA H/ACA |
| ceN86 | 0.160119 | N | 0.265326 | N | snoRNA H/ACA |
| ceN107-3 | 0.159171 | N | 0.126778 | N | SRP RNA |
| ceN92 | 0.098706 | N | 0.175604 | N | snoRNA H/ACA |
| ceN36-1 | 0.065041 | N | 0.218554 | N | snoRNA H/ACA |
| ceN47 | 0.055092 | N | 0.164053 | N | snoRNA C/D |
| ceN80 | 0.044937 | N | 0.315909 | N | snoRNA H/ACA |
| ceN102 | 0.041847 | N | 0.093375 | N | snoRNA H/ACA |
| ceN38 | 0.039686 | N | 0.028512 | N | snoRNA H/ACA |
| ceN79 | 0.035791 | N | 0.025756 | N | snoRNA H/ACA |
| ceN84 | 0.034714 | N | 0.019902 | N | snoRNA H/ACA |
| ceN17 | 0.030383 | N | 0.137699 | N | snoRNA C/D |
| ceN57 | 0.029786 | N | 0.144494 | N | snoRNA C/D |
| ceN94 | 0.029397 | N | 0.213553 | N | snoRNA H/ACA |
| ceN118 | 0.02818 | N | 0.066144 | N | snoRNA C/D |
| ceN65 | 0.027934 | N | 0.056356 | N | snoRNA C/D |
| ceN69 | 0.027758 | N | 0.106046 | N | snoRNA C/D |
| ceN103 | 0.025877 | N | 0.060798 | N | snoRNA C/D |
| ceN104 | 0.025666 | N | 0.0468 | N | snoRNA H/ACA |
| ceN63 | 0.025165 | N | 0.076938 | N | snoRNA C/D |
| ceN127 | 0.024887 | N | 0.242285 | N | snoRNA H/ACA |
| ceN28 | 0.02412 | N | 0.0249 | N | snoRNA C/D |
| ceN10 | 0.023511 | N | 0.049596 | N | RNAase P RNA |
| ceN83 | 0.022838 | N | 0.025485 | N | snoRNA H/ACA |
| ceN4 | 0.021794 | N | 0.553227 | N | snRNA U6 |
| ceN100 | 0.021423 | N | 0.081797 | N | snoRNA H/ACA |
| ceN68 | 0.021187 | N | 0.017192 | N | snoRNA H/ACA |
| ceN61 | 0.021047 | N | 0.061748 | N | snoRNA C/D |
| ceN13 | 0.020943 | N | 0.010547 | N | snoRNA C/D |
| ceN52 | 0.02046 | N | 0.041222 | N |  |
| ceN55 | 0.01944 | N | 0.059602 | N | snoRNA H/ACA |
| ceN54 | 0.01932 | N | 0.064065 | N | snoRNA C/D |
| ceN90 | 0.018794 | N | 0.017274 | N | snoRNA H/ACA |
| ceN111 | 0.01873 | N | 0.020187 | N | snoRNA C/D |
| ceN48 | 0.0187 | N | 0.050887 | N | snoRNA H/ACA |
| ceN34 | 0.018698 | N | 0.468725 | N |  |
| ceN82 | 0.017609 | N | 0.030388 | N | snoRNA H/ACA |
| ceN109 | 0.017022 | N | 0.054217 | N | snoRNA C/D |
| ceN70 | 0.015956 | N | 0.166259 | N | snoRNA C/D |
| ceN49 | 0.015328 | N | 0.032413 | N | snoRNA H/ACA |
| ceN56 | 0.014744 | N | 0.0441 | N |  |
| ceN41 | 0.014716 | N | 0.015319 | N | snoRNA H/ACA |
| ceN101 | 0.014052 | N | 0.008302 | N | snoRNA H/ACA |
| ceN106 | 0.013971 | N | 0.007069 | N | snoRNA C/D |
| ceN58 | 0.013678 | N | 0.025378 | N | snoRNA H/ACA |
| ceN88 | 0.012787 | N | 0.183137 | N | snoRNA H/ACA |
| ceN53 | 0.012437 | N | 0.029397 | N | snoRNA C/D |
| ceN99 | 0.012394 | N | 0.218622 | N | snoRNA H/ACA |
| ceN46 | 0.012232 | N | 0.005888 | N | snoRNA H/ACA |
| ceN44 | 0.011965 | N | 0.043994 | N | snoRNA C/D |
| ceN73-2 | 0.01196 | N | 0.5335 | N |  |
| ceN95 | 0.011938 | N | 0.038516 | N | snoRNA H/ACA |
| ceN125 | 0.011574 | N | 0.030497 | N | snoRNA H/ACA |
| ceN40 | 0.010655 | N | 0.051761 | N | snoRNA C/D |
| ceN42 | 0.010534 | N | 0.006135 | N | snoRNA H/ACA |
| ceN59 | 0.010142 | N | 0.018897 | N | snoRNA H/ACA |
| ceN96 | 0.009995 | N | 0.019793 | N | snoRNA H/ACA |
| ceN110 | 0.009854 | N | 0.007688 | N | snoRNA H/ACA |
| ceN87 | 0.00946 | N | 0.010238 | N | snoRNA H/ACA |
| ceN64 | 0.009359 | N | 0.07464 | N |  |
| ceN9 | 0.009188 | N | 0.01719 | N | scRNA yrn-1 |
| ceN51 | 0.008944 | N | 0.029504 | N | snoRNA H/ACA |
| ceN72 | 0.008677 | N | 0.006765 | N |  |
| ceN97 | 0.00801 | N | 0.023342 | N | snoRNA H/ACA |
| ceN126 | 0.007932 | N | 0.032034 | N | snoRNA H/ACA |
| ceN60 | 0.007579 | N | 0.033823 | N | snoRNA C/D |
| ceN15 | 0.006976 | N | 0.124274 | N | snoRNA C/D |
| ceN89 | 0.00697 | N | 0.078498 | N | snoRNA C/D |
| ceN39 | 0.00686 | N | 0.010283 | N | snoRNA H/ACA |
| ceN124 | 0.00674 | N | 0.033845 | N | snoRNA C/D |
| ceN123 | 0.006518 | N | 0.038611 | N | snoRNA C/D |
| ceN108 | 0.006275 | N | 0.007312 | N | snoRNA C/D |
| ceN43 | 0.006259 | N | 0.001894 | N | snoRNA H/ACA |
| ceN128 | 0.006237 | N | 0.035706 | N | snoRNA H/ACA |
| ceN78 | 0.005977 | N | 0.010522 | N | snoRNA H/ACA |
| ceN62 | 0.005635 | N | 0.015505 | N | snoRNA C/D |
| ceN75 | 0.005209 | N | 0.017079 | N |  |
| ceN93 | 0.005196 | N | 0.062989 | N | snoRNA H/ACA |
| ceN81 | 0.004015 | N | 0.033793 | N | snoRNA H/ACA |
| ceN114 | 0.003384 | N | 0.006369 | N | snoRNA C/D |
| ceN45 | 0.002926 | N | 0.086006 | N | snoRNA H/ACA |
| ceN77 | 0.002766 | N | 0.007642 | N |  |
| ceN71 | 0.002344 | N | 0.008671 | N |  |
